# A new magnetic resonance imaging-based PUMCH classification system for congenital cervical malformations: devising a standardised diagnosis pathway

**DOI:** 10.1186/s13244-024-01708-6

**Published:** 2024-07-18

**Authors:** Zhi-Lin Yuan, Jing Ren, Meng-Lin Huang, Ya-Fei Qi, Xin Gao, Yi-Ying Sun, Yong-Lan He, Lan Zhu, Hua-Dan Xue

**Affiliations:** 1 Department of Radiology, Peking Union Medical College Hospital, Peking Union Medical College and Chinese Academy of Medical Sciences, Beijing, PR China; 2 Department of Obstetrics and Gynecology, Peking Union Medical College Hospital, Chinese Academy of Medical Sciences & Peking Union Medical College, Beijing, PR China

**Keywords:** Congenital cervical malformations, Magnetic resonance imaging, Classification, Diagnosis, Treatment

## Abstract

**Objectives:**

To develop an innovative magnetic resonance imaging (MRI)-based PUMCH (Peking Union Medical College Hospital) classification system aimed at standardising the diagnosis of congenital cervical malformations (CCMs) by identifying their distinctive MRI features.

**Methods:**

Seventy-nine consecutive patients with CCM underwent pre-treatment pelvic MRI; three experienced gynaecological radiologists retrospectively analysed these images. Qualitative assessments included Rock et al’s classification; PUMCH classification; haematometra; cervical signal features; ovarian endometriosis; haematosalpinx; and uterine, vaginal, urinary, and musculoskeletal malformations. Quantitative assessments involved the uterine volume, sagittal cervical length, and maximum ovarian cross-sectional area. The surgical treatment types were also recorded. Statistical methods were used to incorporate differences in clinical features and surgical methods into our classification.

**Results:**

Morphologically, CCMs were categorised into three types: type I (53%) was characterised by the presence of a cervix with visible cervical canals; type II (23%) featured an existing cervix with concealed cervical canals; and type III (24%) indicated cervical aplasia, which involves a blind end in the lower part of the uterine corpus. Haematometra was significantly more prevalent in patients with type I CCM than in those with type II (*p* < 0.001). There were three cervical signal patterns: no signal (27%), no evident layer differentiation (21%), and multi-layer differentiation with haematocele (52%). Most patients (94%) had complete vaginal atresia. Type I CCM patients had a higher likelihood of regaining normal uterovaginal anatomy compared to types II and III.

**Conclusions:**

Our proposed PUMCH classification system has a high potential for enhancing the efficiency of clinical diagnosis among patients with CCM.

**Critical relevance statement:**

The proposed new PUMCH classification promised to elevate the conventional diagnostic trajectory for congenital cervical malformations, offering a valuable framework to refine the selection and planning of surgical interventions, thereby enhancing overall clinical efficacy.

**Key Points:**

- Effective classification of congenital cervical malformations is desirable to optimise the diagnostic process.
- We presented a PUMCH classification of congenital cervical malformations using pelvic MRI.
- The new classification significantly aids clinical triage for congenital cervical malformations.

**Graphical Abstract:**

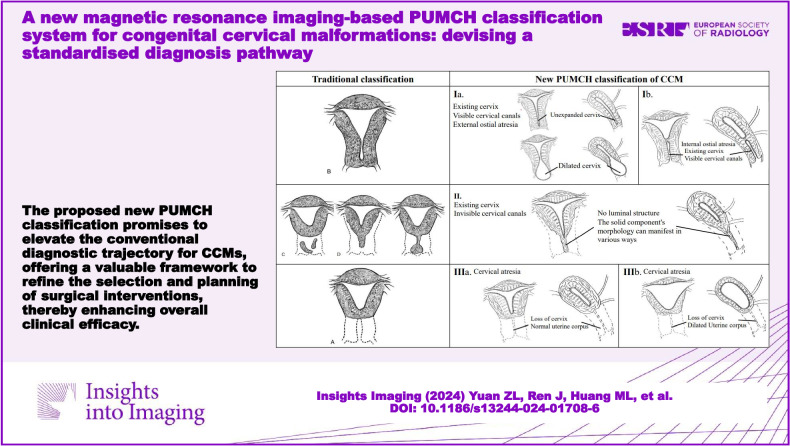

## Introduction

Congenital cervical malformations (CCMs) are rare deformities of the female lower genital tract [1]. These malformations are believed to be caused by the abnormal fusion of the Müllerian ducts and/or lack of subsequent canalisation during development and manifest as the absence or underdevelopment of the cervix. The prevalence of CCMs in the population ranges from 1 to 1.25 cases per 100,000 people, and congenital vaginal agenesis coexists in approximately half of these patients [2]. The likelihood of CCM should be highly suspected in pubertal young women with primary amenorrhoea, cyclical abdominal pain, or a pelvic mass [3]; a lengthy delay in diagnosing this condition (i.e., 1–5 years) often results in the development of endometriosis [4, 5]. Prompt clinical management of CCM is crucial to alleviate any obstructions and prevent severe complications that may lead to irreversible damage to reproductive potential, as such sequelae may require interventions such as salpingectomy and hysterectomy [6, 7].

Hysterectomy has traditionally been a widely accepted and effective treatment option for CCM [8]. Although advances in surgical techniques and reconstructive expertise led to other viable conservative options, such as canalisation [9, 10], such methods may cause serious complications [11, 12]. Currently, treatment selection is primarily based on the type of cervical malformation and presence of the vagina. CCMs are classified as type C4 female genital tract anomalies by the European Society of Human Reproduction and Embryology/European Society for Gynaecological Endoscopy [13] and as type IB congenital anomalies by the American Fertility Society/American Society for Reproductive Medicine [14]. Although the most widely accepted system is that of Rock et al, which is based on a study of 30 patients, there remains ongoing controversy regarding the clinical practicality of CCM subclassifications [8].

Magnetic resonance imaging (MRI) is a non-invasive tool that is routinely used to diagnose CCM owing to its high soft tissue contrast and multiplanar imaging capability [15, 16]. This imaging modality provides high-quality and accurate anatomical information, thereby aiding in the early diagnosis of cervical malformations [17]. However, Rock et al’s subclassification primarily relies on anatomical structures; this necessitates confirmation via postsurgical evaluation and creates a challenge in terms of detecting anomalies based solely on imaging tests [18]. To date, a comprehensive subclassification of MRI-based morphological and signal features in a statistically significant number of patients is lacking. Hence, there is an unmet need for a widely accepted and practically employable CCM classification system that can guide appropriate surgical management [19].

The main goal of this study was to devise a new and effective classification system of CCM aimed at standardising the diagnostic process for this condition. To that end, we aimed to explore the unique MRI characteristics of these malformations.

## Methods

### Patients

**Fig. 1:**
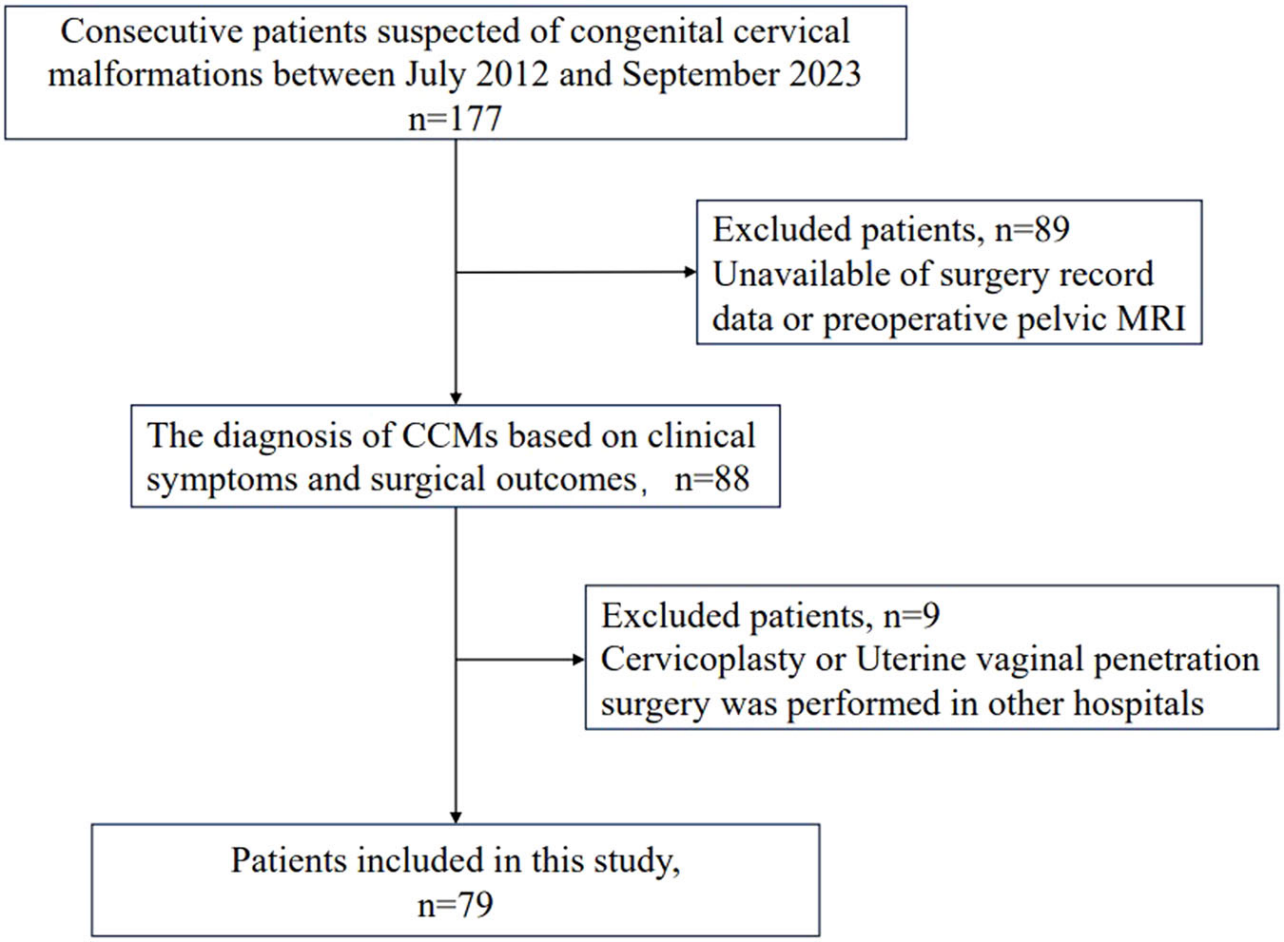
Flowchart of the patient inclusion and exclusion criteria used in this study

The institutional review board of Peking Union Medical College Hospital (PUMCH) approved this retrospective study and waived the requirement for informed consent. The study included patients who were treated for CCM at our institution between July 2012 and September 2023. The inclusion criteria were (i) availability of preoperative pelvic MRI and surgery record data and (ii) diagnosis of CCM based on clinical syndromes and surgical outcomes. The exclusion criterion was cervicoplasty or uterine vaginal penetration surgery performed at other hospitals. Ultimately, 79 consecutive patients with a mean age of 14.5 ± 3.6 years were included in this study (Fig. 1).

### MRI acquisition

MRI was performed using 3.0-T MR (13 patients: Siemens Skyra, 10 patients: GE Medical Systems Signa PET/MR, nine patients: Philips Ingenia Elition X, eight patients: GE Medical Systems Discovery MR 750w, 12 patients: Siemens Magnetom Vida, one patient: TOSHIBA Titan 3 T, and one patient: GE Medical Systems Discovery MR 750) or 1.5-T (17 patients: GE Medical Systems Signa Excite, four patients: GE Medical Systems Signa HDxt, two patients: TOSHIBA_MEC MRT200SP5, one patient: GE Medical Systems Brivo MR355, and one patient: GE Medical Systems Optima MR360) imagers with a body-array coil. Scanning was performed with the patient in the supine position. A detailed overview of the MRI parameters is shown in Table 1. At a minimum, axial T1-weighted images (T1WIs), as well as axial, sagittal, and coronal T2-weighted images (T2WIs) were obtained [20]. Given the retrospective design of the study, no significant modifications were made to the MRI sequences or parameters. There was no utilisation of any antispasmodics throughout the entire procedure. Data from patients were analysed insofar as the image quality was sufficient for diagnosis, as the primary focus of the study was determining the anatomical features. All patients underwent surgical intervention within a period of two months after MRI.

**Table 1:** MR imaging parameters details

Parameters	T1-weighted	T2-weighted	T2-weighted	T2-weighted
Imaging acquisition	TSE/FRFSE	TSE/FRFSE	TSE/FRFSE	TSE/FRFSE
Orientation	Axial	Axial	Sagittal	Coronal
Repetition time/echo time (ms)	125–800/4–20	2993–9744/77–138	2940–8565/85v136	900–7321/86–136
Field of view (mm^2^)	304 × 250	304 × 250	300 × 300	400 × 400
Slice thickness (mm)	4–8	4–8	3–7	4–6
No. of slices	16–24	16–24	16–24	16–24

### Image analysis

Pelvic MRIs were independently analysed in a randomised order by two experienced gynaecological radiologists (Z.Y. and J.R.) with three and six years of experience in MRI interpretation, respectively. The radiologists were blinded to patients’ pathological information, and all features that required evaluation were pre-described. After the initial reading, a conference was held to review all the cases, and any discrepancies were arbitrated by a senior radiologist (Y.H.) with 13 years of experience in gynaecological MRI interpretation. The basic MRI-based characteristics are shown in Table 2.

**Table 2:** Assessment of basic characteristics on MR images

	Assessment content of CCMs on MR images
**Qualitative assessments**	Rock’s classification (aplasia/obstruction/fibrous cord/fragmentation)
	PUMCH classification (type I/type II/type III)
	Uterine malformations (existence/inexistence)
	Haematometra (existence/inexistence)
	Cervical signal features (no signal/no-evident layer/multiple-evident layer)
	Ovarian endometriosis (existence/inexistence)
	Haematosalpinx (existence/inexistence)
	Vaginal malformation (complete agenesis/incomplete agenesis)
	The malformation of the urinary and skeletal system (existence/inexistence)
**Quantitative assessments**	Uterine volume (mL)
	Sagittal length of cervix (cm)
	Maximum cross-section area of ovary (cm^2^)

**Fig. 2:**
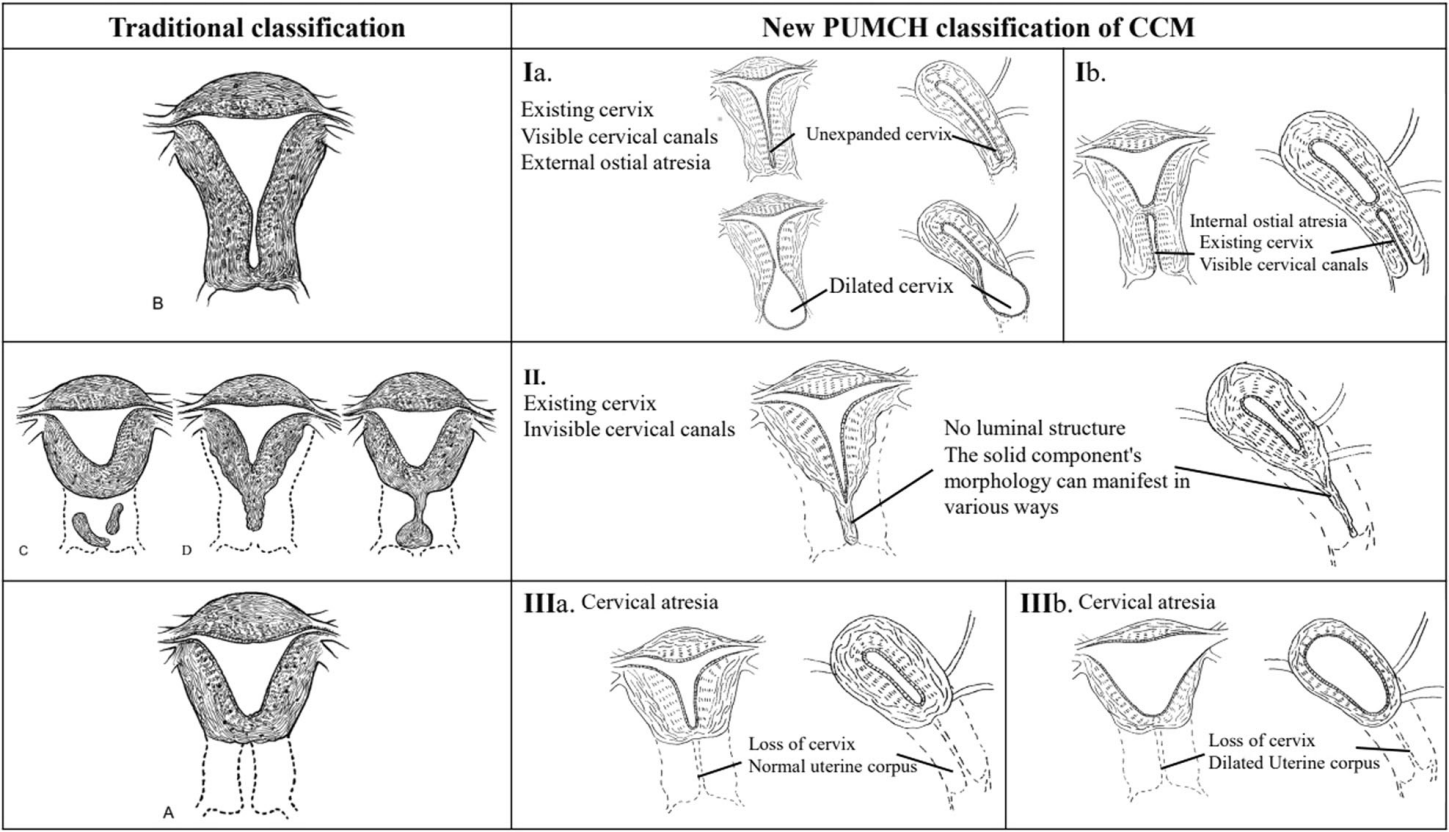
Comparative anatomical diagrams depicting the traditional classification for abnormal cervical development alongside the proposed typing for cervical atresia (coronal and sagittal views of the uterus)

Qualitative assessments: Rock et al’s classification of each patient was recorded; if any patient could not be classified, the reason for this was noted. We proposed a PUMCH classification in which patients were categorised into three subgroups depending on the morphology of the cervix. These subgroups were visualised on MRI scans as follows (Fig. 2): Type I referred to an existing cervix with a visible cervical canal and was further subclassified into internal versus external ostial atresia. Type II denoted an existing cervix with unobservable cervical canals. Type III referred to cervical aplasia (blind end of the lower part of the uterine corpus) and was further subclassified into dilated versus non-dilated uterus. According to the four-layer band-like structure can be seen in normal cervi on MR imaging [21], the signal manifestation of the malformed cervix was generally classified into three types: no signal (no detection of cervix), no-evident layer differentiation, and multiple-evident layer differentiation with a haematocele signal inside the cervical canal. Signal intensity was categorised as low, moderate, or high based on normal myometrium signals on T1WI and T2WI. Additionally, urinary system malformations that primarily encompassed kidney deformities and anal atresia were recorded, as were musculoskeletal malformations (predominantly involving spinal deformities).

Quantitative assessments: Uterine volume was determined based on the formula used for an ellipsoid [22]. The sagittal length of the malformed cervix was measured from the top of the vagina to the bottom of the uterine corpus. The maximum ovarian cross-sectional area was calculated using the formula for an ellipse; however, ovaries with endometriosis were not evaluated. All measurements were independently acquired by two radiologists and averaged.

### Clinical data collection

Detailed clinical characteristics and pathological information were obtained from the electronic medical records at our institution and meticulously reviewed. The type of surgical procedure undergone by each patient was noted. Surgery was divided into two types: restoration of normal uterovaginal anatomical structure and hysterectomy. Patients who underwent vaginoplasty, cervicoplasty, or uterine vaginal penetration surgery were categorised as the former type. Furthermore, pertinent clinical information, including pelvic adhesions and reasons for the failure of conservative surgery, were collected from patients’ surgical records.

### Statistical analysis

Statistical analyses were conducted using SPSS version 26.0.0.0 (IBM Corp., Armonk, NY, USA). Qualitative variables are expressed as frequencies. The Shapiro-Wilk test was used to test the normality of continuous variables, which are expressed as the means ± standard deviations. The chi-square, Fisher’s exact (qualitative variables), or Kruskal–Wallis tests (quantitative variable) were used to compare patients of different morphological groups. A double-sided *p* value < 0.05 was considered statistically significant.

## Results

### MRI characteristics of the different CCM types

The pelvic MRI characteristics in patients of different morphology groups are summarised in Table 3. Haematometra was more common in patients with type I CCM than in those with type II CCM (*p* < 0.001). Moreover, the mean sagittal length was longer in patients with type I CCM than in those with type II CCM (*p* < 0.001). No significant differences were observed between the different groups with respect to other MRI features such as uterine volume, haematosalpinx, and degree of vaginal dysgenesis (*p* > 0.05). The detailed MRI findings of the entire patient population are described below.

**Table 3:** MRI characteristics and operative treatments of CCMs patients in different types

PUMCH classification	Type I (*n* = 42,53%)	Type II (*n* = 18,23%)	Type III (*n* = 19,24%)	*p* value
MRI Characteristics				
Uterus				
Malformation, *n* (%)	7 (16.7)	1 (5.6)	8 (42.1)	0.033
Haematometra, *n* (%)	41 (97.6)^‡^	10 (55.6)^‡^	16 (84.2)	< 0.001
Volume (mL), mean (SD)	63.3 (40.7)	40.8 (15.4)	85.0 (56.9)	0.050
Cervix				
No signal, *n*	/	/	19	
No-evident layer, *n*	/	18	/	
Multiple-evident layer, with haematocele, *n*	42	/	/	
Sagittal length (cm), mean (SD)	5.6 (3.2)	1.9 (0.6)	/	< 0.001
Ovary				
Maximum cross-section area (cm^2^), mean (SD)^†^	4.0 (2.1)	5.7 (4.3)	4.4(1.9)	0.095
Ovarian endometriosis, *n* (%)	7 (16.7)	6 (33.3)	7 (36.8)	0.147
Haematosalpinx, *n* (%)	15 (35.7)	6 (33.3)	8 (42.1)	0.842
Vagina*				0.375
Complete agenesis, *n*	38	17	19	
Incomplete agenesis, *n*	3	1	0	
Urinary system				
Renal malformation, *n* (%)	4 (9.5)	1 (5.6)	0	/
Anal atresia, *n* (%)	2 (4.8)	1 (5.6)	3 (15.8)	/
Skeletal system				
Spinal deformity, *n* (%)	3 (7.1)	1 (5.6)	2 (10.5)	/
Operative treatments				< 0.001
Hysterectomy, *n*	6	13	11	
Restore normal anatomy, *n*	36	5	8	

The findings in patients of different morphological types were analysed with the chi-square test, Fisher’s exact test (qualitative variables) or Kruskal-Wallis test (quantitative variables); /, no patients; n, number of patients; ^‡^The differences were only found in these two types; ^†^Ovaries were not measured if there exists ovarian endometriosis; *One patient had a normal vagina

#### Cervix

**Fig. 3:**
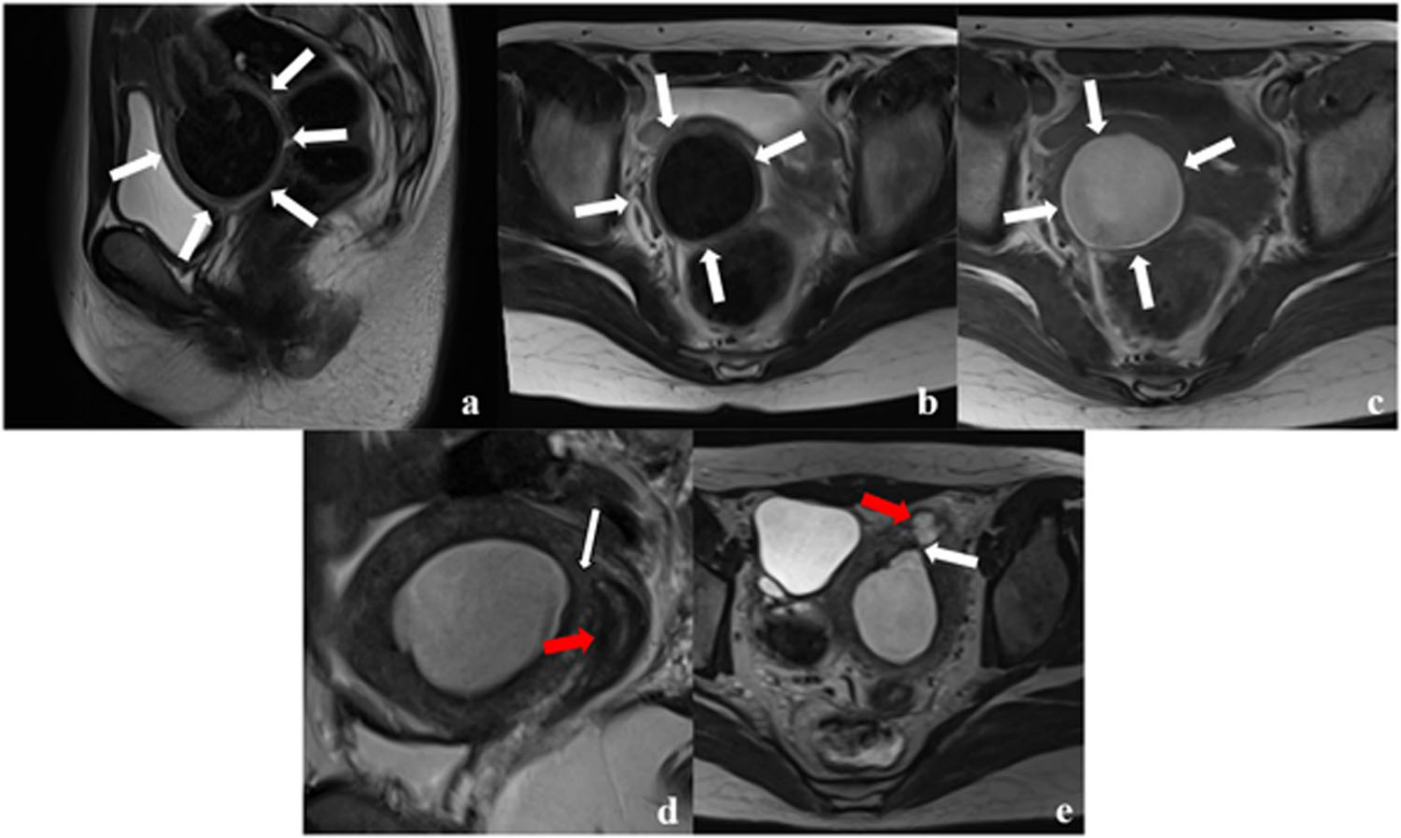
Magnetic resonance images of different subgroups of patients with type I congenital cervical malformations. Sagittal T2-weighted (**a**) and axial T2-weighted (**b**) and T1-weighted (**c**) images acquired from a 12-year-old patient with external ostial atresia. Sagittal and axial T2-weighted images reveal an enlarged cervix (white arrows) with a multi-layered differentiation signal, demonstrating haematocele presence, while T1-weighted images exhibit a prominent hyperintense signal in the cervix (white arrows). Sagittal T2-weighted (**d**) and axial T2-weighted (**e**) images acquired from a 13-year-old patient with internal ostial atresia. Sagittal and axial T2-weighted images show internal ostial atresia (white arrows), while the cervical canal exhibits a multi-layered differentiation signal (red arrows)

**Fig. 4:**
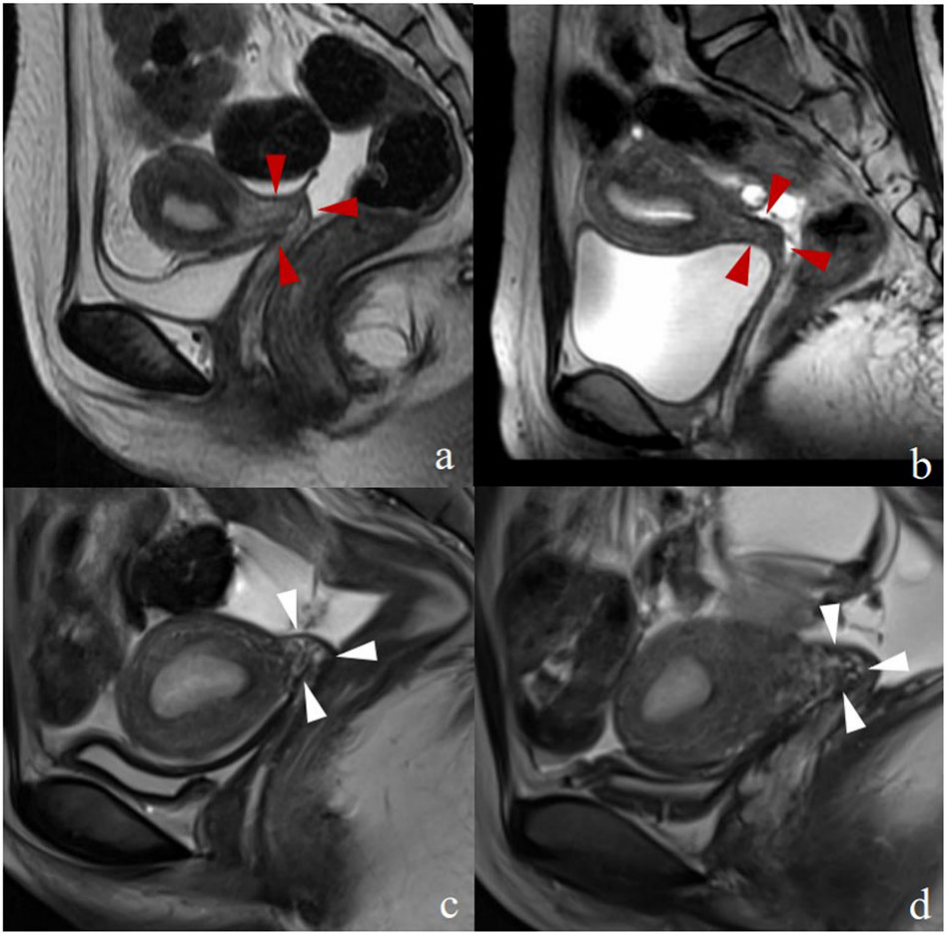
Magnetic resonance images depicting four individuals classified as having type II congenital cervical malformations. The sagittal T2-weighted images of these patients revealed the absence of luminal structures. Among these, two displayed homogeneous signals (red arrowheads in **a** and **b**), while the remaining two exhibited conspicuous mixed signals (white arrowheads in **c** and **d**)

**Fig. 5:**
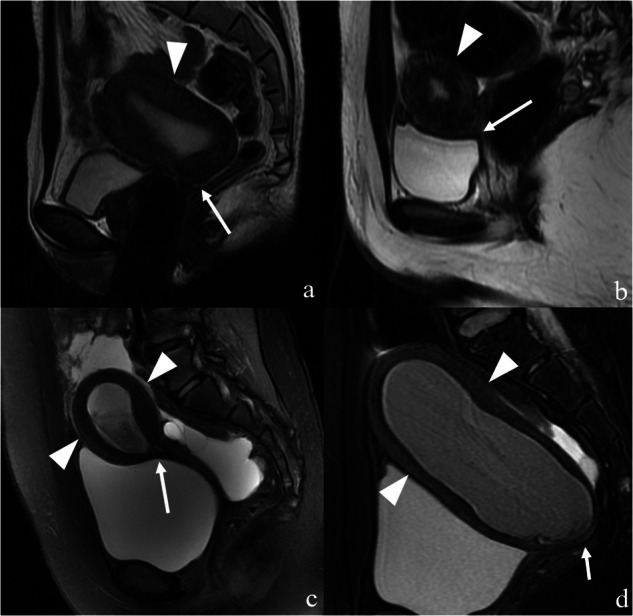
Magnetic resonance images illustrating two subgroups of patient with type III congenital cervical malformations. The images clearly depict the normal body of the uterus (white arrowheads in **a** and **b**) along with the absence of the cervix (white arrows in **a** and **b**). Some patients exhibited an evident abnormal uterine corpus characterised by numerous haemorrhage signals (white arrowheads in **c** and **d**) as well as a missing cervix (white arrows in **c** and **d**)

Morphologically, the cervixes were categorised into three general types. Type I denoted a cervix with cervical canals (42 patients; 53%) and was further subdivided into internal ostial atresia (Fig. 3a–c) and external ostial atresia (Fig. 3d, e). Type II (18 patients; 23%) was marked by a cervix with invisible cervical canals, displaying homogeneous solid isointensity compared to the outer myometrium [23] on the same image slice (Fig. 4a, b), or displaying mixed signal (Fig. 4c, d). Type III (19 patients; 24%) was defined by a blind end in the lower part of the uterine corpus. Some patients exhibited a normal uterine morphology (Fig. 5a), while others showed abnormalities marked by numerous haemorrhage signals in the dilated uterine cavity (Fig. 5b). Notably, patients with type I tended to have longer cervixes than those with type II (5.6 ± 3.2 vs. 1.9 ± 0.6 cm, *p* < 0.001).

The signal-based features of the cervix were generally classified into three types: no signal (19 patients; 24%) (Fig. 5), no-evident layer differentiation (18 patients; 23%) (Fig. 4), and evident layer differentiation with or without a haematocele signal inside the cervical canal (42 patients; 53%) (Fig. 3). Clear layer differentiation within the cervix was common among patients with type I, and was often accompanied by a haematocele signal inside the cervical canals. Within this group, 45.2% of patients displayed three-layer differentiation, while 54.8% exhibited four-layer differentiation. On the other hand, patients with type II typically lacked evident layer differentiation within the cervix, and those with type III showed no signal owing to the blind end in the lower part of the uterine corpus.

Our patients were also classified according to Rock et al’s system based on MRI findings. Nineteen patients had cervical aplasia, while 41 had cervical obstruction; moreover, 15 had a fibrous cord. However, it was challenging to apply Rock et al’s classification to four of the patients. One patient did not exhibit cervical aplasia or obstruction but merely had an internal ostium atresia (PUMCH classification: type Ib). The remaining three each had a small cervix that did not align with the classification criteria (PUMCH classification: type II, they do have a cervix but invisible cervical canals on MR images). None of the patients were diagnosed with cervical fragmentation.

#### Uterus

Uterine malformations were detected in 16 patients; these included uterine agenesis (two; 12.5%), bicornuate uterus (one; 6.2%), uterus duplex (three; 18.8%), complete septate uterus (two; 12.5%), incomplete septate uterus (two; 12.5%), and rudimentary horn uterus (six; 37.5%). Haematometra, characterised by bleeding within the uterine cavity accompanied by dilatation thereof, was observed in 67 patients. Among those, haemorrhaging was observed in 41 patients with type I CCM, 10 with type II, and 16 with type III (*p* < 0.001). The median uterine volume among patients with type I CCMs was 63.3 ± 40.7 mL, while the median volumes in patients with types II and III were 40.8 ± 15.4 and 85.0 ± 56.9 mL, respectively. However, these differences were not statistically significant (*p* = 0.050). Additionally, adenomyosis was present in three of the patients in this study, all of whom were type II.

#### Adnexa

The maximum cross-sectional areas of the ovaries of patients with types I, II, and III CCM were 4.0 ± 2.1, 5.7 ± 4.3, and 4.4 ± 1.9 cm², respectively (*p* = 0.095). Ovarian endometriosis was diagnosed in 20 patients, with bilateral involvement in 10, right ovary only in four, and left ovary only in six. Additionally, the incidence of endometriosis did not differ significantly among patients with different types of CCM (*p* = 0.147). Pelvic endometriosis was detected in one patient, predominantly located in the posterior wall of the uterus, uterorectal fossa, fundal ligament, left ovary, and fallopian tube. Haematosalpinx was identified in 29 patients, including 15 with type I, six with type II, and eight with type III (*p* = 0.842); this condition typically presented as a tortuous and dilated fallopian tube that showed high signal intensity on T1WI and variable signal intensity on T2WI (i.e., either high or low).

#### Vagina

The distal vagina was discernible on MRI in only 28 of the 79 patients. Most patients (74; 94%) also had complete vaginal atresia, including 38 with type I, 17 with type II, and 19 with type III (*p* = 0.661). One patient had a normal vagina.

#### Urinary and skeletal system

Six patients exhibited congenital spinal deformity, while another five presented with congenital renal malformations that included conditions such as renal agenesis and duplication. Additionally, six patients had a documented history of congenital anal atresia.

### Related clinical characteristics

The surgical methods undergone by the patients were classified into two groups. The first was the hysterectomy group that included 30 patients; six had type I CCM, 13 had type II, and 11 had type III. Of the six patients with type I, two had uterine malformations and four opted for total hysterectomy owing to thick or poor interstitial tissue. In contrast, patients with types II and III predominantly underwent hysterectomy (*p* < 0.001), primarily owing to anatomical factors such as the greater distance from the vaginal acupoint to the uterine corpus or the extremely thin space between the rectum and bladder. These anatomical considerations likely rendered other treatment options less feasible or effective. The other surgical group comprised 49 patients who underwent procedures aimed at restoring normal uterovaginal anatomical structure, including uterovaginal anastomosis or cervicoplasty. This group comprised six patients with type I CCM, five with type II, and eight with type III. Furthermore, pelvic adhesions were discovered in 57 patients (27, 15, and 15 with types I, II, and III, respectively; *p* = 0.266).

## Discussion

In this study, we comprehensively assessed pelvic MRI characteristics and developed a new radiological PUMCH classification for CCMs using a cohort of 79 patients, which has classified patients with CCMs into three types. Furthermore, our findings indicate that patients diagnosed with type I CCM have a higher likelihood of restoring normal reproductive anatomy compared to individuals with types II and III. The three proposed classifications are potentially highly useful for clinicians to understand the anatomical structure of patients and maximise the standardisation of diagnosis and treatment.

We classified patients with CCM into three types based on MRI data. Type I includes those with a cervix present and visible cervical canals, type II comprises those with a present cervix but with invisible cervical canals, and type III includes those with cervical aplasia characterised by a blind end at the lower part of the uterine corpus. A normal cervix appears as a four-layer band-like structure on imaging; the multiple-layer band-like differentiation observed in patients with type I CCM was similar to that of a normal cervix, which was consistent with a previous study [2, 24]. This implies that patients with type I may have structural cervix atresia yet retain some functionality, whereas those with types II and III are more inclined to experience both functional and structural irregularities. This finding might explain the higher incidence of haematometra in patients with type I than in those with type II, as the development of the cervical canal is closely related to that of the endometrium [25, 26]. However, no significant differences were observed between types I and III. It may be necessary to increase the number of patients across all types in future studies to obtain conclusive results. Additionally, endometriosis was found in 22% of patients with CCMs, which is higher than the reported rates for women of reproductive age (10%) [27] and consistent with data from previous studies [4, 28]. At the same time, the incidences of genitourinary and musculoskeletal malformations in patients with CCM were higher than the normal rates of 0.1% and 1–3%, respectively, which was consistent with other studies [19, 29, 30].

Previous investigations of CCMs were limited in terms of detailed and comprehensive descriptions of structural and imaging features, and specifically lacked large cohort-based MRI analyses [2, 4, 8, 19, 24]. Moreover, the majority of previously published female genital tract malformation classification systems did not include specific subclassifications for the cervix [14, 15, 31–34]. Xie et al [35] categorised cervical malformations into four distinct types based on a combination of anatomical and ultrasonographic findings. However, their small sample size and the limited ability of ultrasonography to accurately assess anatomical structures posed challenges in terms of the application of this system in clinical practice. Rock et al’s classification stands as a widely accepted method for categorising CCMs, offering a comprehensive breakdown of their anatomical structure. Building upon this foundational anatomical framework, we introduced a novel PUMCH classification, which further integrated additional MR imaging features and offered a more nuanced subclassification. Details of the advantages and limitations of Rock et al’s classification and the proposed PUMCH classification were listed in Table 4. Rock et al’s classification divided CCMs into four categories: cervical aplasia, cervical fragmentation, fibrous cord, and cervical obstruction. However, this classification requires confirmation via invasive methods such as exploratory laparotomy or post-surgical evaluation; moreover, assessing this classification using MRI can be challenging in certain cases [8, 36]. Other previous studies barely considered the use of MRI for identifying cervical fragmentation [18, 37]. This was evident in our study, as none of our patients were diagnosed with cervical fragmentation using MRI. Additionally, applying Rock et al’s classification using MRI data was challenging in four of our patients. The primary challenge could stem from the inability to apply the actual anatomy to any of the criteria for Rock et al’s classification.

**Table 4:** Comparison of Rock et al’s classification and PUMCH classification

	Rock et al’s classification			PUMCH classification
Advantages	Summarising the anatomic types of cervical malformations.		a. Comprehensively summarizing the anatomical structure and MR imaging features of cervical malformations. b. Beneficial to the clinical diagnosis and treatment of CCM patients. c. Simple and easy to classify, suitable for all present CCM patient.	
Limitations	a. Surgery is needed for accurate typing. b. Some types (such as cervical fragmentation) cannot be accurately described on imaging. c. Based on only 30 patients.	a. Patient data came from a single centre only.		

Our proposed PUMCH classification is potentially helpful for devising clinical treatment plans for patients with CCM. Patients with type I CCM are more likely to regain normal reproductive anatomy than those with types II and III and may be advised to undergo conservative surgery such as uterine vaginal penetration [9, 10]. Patients with type II CCM had cervixes that usually appeared solid without a luminal component; for such patients, the use of canalisation and cervical reconstruction to restore anatomical function is often considered [38]. Patients with type III CCM typically undergo uterine vaginal anastomosis or cervical reconstruction to restore the reproductive tract structure owing to the blind end below the corpus uteri [10, 39, 40]. Nevertheless, conservative surgery may more frequently fail among patients of the latter two types owing to the thickness of the tissue, longer distance between the uterine cavity and uterus, or narrow gap. Furthermore, to provide optimal support for clinicians, radiologists should emphasise key imaging features when managing patients with CCM. These essential features encompass PUMCH classification, assessment of uterine malformations and haematometra, evaluation of endometriosis and pelvic adhesions, detection of haematosalpinx, identification of malformations of the genitourinary and musculoskeletal systems, and recognition of vaginal atresia.

The present study had certain limitations. First, it was a retrospective, observational study conducted at a single centre; therefore, it was inevitably prone to sampling biases. Second, the number of included patients was limited. Although CCMs are rare, gathering larger samples from several different centres would help further refine this classification system. Third, the lack of respectively oblique axial and oblique coronal sequences was a limitation of this study, which could potentially impact the assessment of certain imaging features. To address this, we plan to incorporate these sequences in future studies to enhance the comprehensiveness of our research. Additionally, we recognise that the slice thickness exceeding 4 mm presents a constraint, potentially influencing the evaluation of some certain structures. Consequently, it will be crucial to consider evaluating thinner slices in future investigations. In summary, we have introduced a novel PUMCH classification for CCMs that categorises patients into three distinct types. We hope that our findings are instrumental in delineating the essential MRI-based characteristics that underlie CCMs. By offering a refined classification framework and comprehensive insight into key MRI features, clinicians can streamline the CCM clinical diagnosis pathway, potentially expediting treatment decisions and ultimately enhancing patient outcomes.

## Data Availability

All data and material were obtained from the Peking Union Medical College Hospital.

## Abbreviations

CCMs: Congenital cervical malformations
MRI: Magnetic resonance imaging
PUMCH: Peking Union Medical College Hospital
T1WI: T1-weighted images
T2WI: T2-weighted images
